# In Vitro and In Vivo Biological Activity of Ruthenium 1,10-Phenanthroline-5,6-dione Arene Complexes

**DOI:** 10.3390/ijms232113594

**Published:** 2022-11-06

**Authors:** Oscar A. Lenis-Rojas, Catarina Roma-Rodrigues, Beatriz Carvalho, Pablo Cabezas-Sainz, Sabela Fernández Vila, Laura Sánchez, Pedro V. Baptista, Alexandra R. Fernandes, Beatriz Royo

**Affiliations:** 1 ITQB NOVA, Instituto de Tecnologia Química e Biológica António Xavier, Universidade Nova de Lisboa, Av. da República, 2780-157 Oeiras, Portugal; 2 Associate Laboratory i4HB—Institute for Health and Bioeconomy, NOVA School of Science and Technology, NOVA University Lisbon, 2819-516 Caparica, Portugal; 3 UCIBIO—Applied Molecular Biosciences Unit, Department of Life Sciences, NOVA School of Science and Technology, NOVA University Lisbon, 2819-516 Caparica, Portugal; 4 Departamento de Zoología Genética y Antropología Física, Facultad de Veterinaria, Universidad de Santiago de Compostela, Campus de Lugo, 27002 Lugo, Spain; 5 Preclinical Animal Models Group, Health Research Institute of Santiago de Compostela (IDIS), 15706 Santiago de Compostela, Spain

**Keywords:** zebrafish, Ru-dione complexes, anticancer activity, autophagy, ROS

## Abstract

Ruthenium(II) arene complexes exhibit promising chemotherapeutic properties. In this study, the effect of the counter anion in Ru(II) complexes was evaluated by analyzing the biological effect of two Ru(II) *p*-cymene derivatives with the 1,10-phenanthroline-5,6-dione ligand of general-formula [(η^6^-arene)Ru(L)Cl][X] X = CF_3_SO_3_ (JHOR10) and PF_6_ (JHOR11). The biological activity of JHOR10 and JHOR11 was examined in the ovarian carcinoma cell line A2780, colorectal carcinoma cell line HCT116, doxorubicin-resistant HCT116 (HCT116-Dox) and in normal human dermal fibroblasts. Both complexes JHOR10 and JHOR11 displayed an antiproliferative effect on A2780 and HCT116 cell lines, and low cytotoxicity in fibroblasts. Interestingly, JHOR11 also showed antiproliferative activity in the HCT116-Dox cancer cell line, while JHOR10 was inactive. Studies in A2780 cells showed that JHOR11 induced the production of reactive oxygen species (ROS) that trigger autophagy and cellular senescence, but no apoptosis induction. Further analysis showed that JHOR11 presented no tumorigenicity, with no effect in the cellular mobility, as evaluated by thye wound scratch assay, and no anti- or pro-angiogenic effect, as evaluated by the ex-ovo chorioallantoic membrane (CAM) assay. Importantly, JHOR11 presented no toxicity in chicken and zebrafish embryos and reduced in vivo the proliferation of HCT116 injected into zebrafish embryos. These results show that these are suitable complexes for clinical applications with improved tumor cell cytotoxicity and low toxicity, and that counter-anion alteration might be a viable clinical strategy for improving chemotherapy outcomes in multidrug-resistant (MDR) tumors.

## 1. Introduction

Nowadays, cancer is, without any doubt, affecting people in every country of the world, with an incidence and mortality rate which places it as the second leading cause of death worldwide [1]. Besides these considerations, it is important to emphasize the fact that multidrug resistance (MDR) continues to be the principal limiting factor to achieving cures in patients with cancer [2], since it is responsible for over 90% of deaths in cancer patients receiving traditional chemotherapeutics or novel targeted drugs [3]. However, and in spite of the advances made regarding new treatments capable of overcoming resistance [2], the most promising option for cancer treatment today is chemotherapy [4]. Platinum drugs are one of the most used chemotherapeutic regimens, which are administered to 50–70% of cancer patients, either as single drugs or in combination with other drugs [5]. Nevertheless, resistance [6] and side effects [7] severely limit the clinical application of platinum-based treatments, and consequently, the search for alternatives is one of the most active areas of inorganic medicinal chemistry.

Since the anticancer properties of 1,10-phenanthroline-5,6-dione (phendione) have become known [8], several metal compounds containing phendione as a ligand have been synthesized with the aim of seeking alternatives to cisplatin. Metal complexes of Cu(II), Ag(I) [8], Au(III) [9], Pt(II) [9,10], Pd(III) [11], Re(I) [12], Ru(II) [13], Co(III/II) and Zn(II) [14]-bearing phendione have been synthetized and tested against renal A-498 and hepatic Hep-G2 breast MCF-7, EVSA-T, colon WIDR, melanoma M19-MEL, renal A498, non-small-cell lung H226, ovarian IGROV-1, and human cancer cell lines, among others. Some of these metal complexes, e.g., [Cu(phendione)_3_](ClO_4_)_2·_4H_2_O, [Ag(phendione)_2_]ClO_4_, [Co(Cl)(phendione)_2_(H_2_O)][BF_4_], and [Zn(phendione)_2_]Cl_2_ displayed higher activity, which resulted in greater activity than cisplatin. However, they displayed limited selectivity, being also active against human non-tumorigenic cell lines as it occurs with the phendione itself [8,9,14]. For [Ru*p*-cymene(phendione)][PF_6_] and [Pt(phendione)Cl_2_], only the cytotoxicy against cancer cells was studied [10,13], and so it was not possible to determine the selectivity to cancer cells. Notably, in some cases, the high cytotoxicity of the phendione ligand was reduced when the ligand was coordinated to a metal center, as in [Re(CO)_3_(phendione)Cl] [12] and [Pd(phendione)(iso-pentylglycine)][NO_3_] [11].

Notable, complexes [Co(Cl)(phendione)_2_(H_2_O)][BF_4_] and [Zn(phendione)_2_]Cl_2_, were active against colon-resistant cancer cells, and the mechanism of action of both complexes was independent of P-glycoprotein (P-gp) activity which enabled them to trigger pathways and to surpass resistance [15,16]. Additionally, the [Pt(phendione)Cl_2_] complex was also active against human ovarian carcinoma, as well as bring sensible and resistant to cisplatin A2780, A2780R [10]. Thus, these results indicate the potential of the phendione complexes for overcoming MDR in cancer. However, one of the most important concerns about this kind of complex is its toxicity, as well as the loss of activity when it coordinates with the metal center.

Our team has recently disclosed the anticancer activity of ruthenium arene complexes bearing N,N-chelating ligands, which were active against several cancer cell lines such as ovarian A2780 and colon HCT116 cancer, with low toxicity or being non-toxic both in vitro and in vivo [17,18]. Due to the anticancer activity and the low toxicity values that we have obtained with the ruthenium arene tetrazine [17], quinoxaline [17], and triazole [18] compounds, as well as inorganic ruthenium polypyridyl complexes [19,20], we decided to explore the anticancer activity of related Ru compounds containing the phendione ligand against resistant cancer lines, namely complexes [(η^6^-arene)Ru(phendione)Cl][X] containing two different counter anions, X = CF_3_SO_3_ (JHOR10) and PF_6_ (JHOR11). The counter anion may have the ability to exert an influence in the internalization rate of the complex, improving the antiproliferative activity with changes to the cell cycle, apoptosis, ROS and mitochondrial membrane potential in cancer cells. Thus, the counter-anion effect may provide an alternative and effective strategy to enhance the anticancer activity for these complexes [21]. Cytotoxicity studies were carried out in vitro in an A2780 cancer cell line, and the in vivo toxicological analysis was performed using zebrafish as a model for toxicity, and for the efficacy in vivo through HCT116 colon cancer. Interestingly, the complex JHOR11 was active against colon cancer and colon cancer-resistant cells. Furthermore, the mechanism of cell death cancer cells was studied, and the colon cancer xenograft has been investigated, showing promising results.

## 2. Results and Discussion

### 2.1. Synthesis

**Scheme 1:**
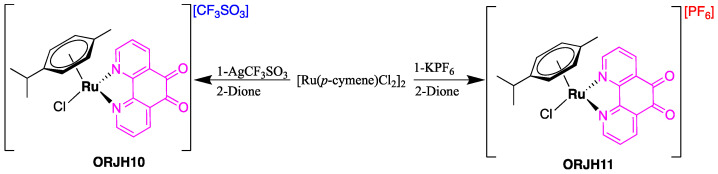
Synthesis of complexes JHOR10 and JHOR11.

The new ruthenium complex JHOR10 was synthesized by the reaction of the Ru(II) precursor [Ru(*p*-cymene)Cl_2_]_2_ with a silver triflate in dichloromethane under the exclusion of light, followed by the addition of one equivalent of 1,10-phenanthroline-5,6-dione as shown in Scheme 1. Complex JHOR11 has been already described and was prepared following the literature procedure [22] (Scheme 1).

The omplex JHOR10 was fully characterized by IR, ^1^H and ^13^C NMR spectroscopies, mass spectrometry and elemental analysis (see Supplementary Materials Figures S1–S3). As expected, the ^1^H NMR spectrum displayed two doublets at 9.56, 8.68 ppm and one doublet of doublets at 7.96 ppm, which corresponded to the CH protons of the pyridine fragments. The spectrum also displayed two doublets at ca. 6.00 ppm of the CH protons of the *p*-cymene moiety, along with the corresponding resonances of the isopropyl group (at 2.74 and 1.11 ppm) and methyl group (2.27 ppm) of the *p*-cymene unit. In addition, high-resolution mass spectrometry (HRMS) showed the expected peak of the molecular fragment [Ru(*p*-cymene)(Phendione)Cl]^+^ in the positive mode.

### 2.2. Complexes Stability

To evaluate the stability of complexes in a cell culture medium, powdered complexes were first solubilized in DMSO and then immediately in DMEM, in order to guarantee that no more than 0.1% (*v*/*v*) DMSO was present in the medium. After 24 h and 48 h incubation at 37 °C, the spectra were acquired for each complex. Results show that both complexes have two peaks at 300 nm and 330 nm (Supplementary Information Figure S4), with no significant alterations occurring during the time course of the experiment, suggesting that complexes are stable in the cell culture medium used in biological analysis. In addition, the stability of the complexes in solution was investigated by ^1^H NMR spectroscopy. The ruthenium complexes were dissolved in DMSO-d_6_-D_2_O, in a 3:7 ratio (*v*/*v*) with a 110 mM chloride concentration, and the ^1^H NMR spectra was recorded immediately and after 48 h. It was observed that under these conditions, new sets of resonances appeared in the spectrum (t = 0 h) of the complex JHOR10 (Supplementary Information Figure S6), indicating the formation of a new species that most probably corresponds to the aqua compounds. This observation is in agreement with the M–Cl aquation processes described for several metallic compounds, such as cisplatin [23]. After 48 h, the ^1^H NMR spectrum of JHOR10 was unchanged. In contrast, complex JHOR11 seems to be more stable under the conditions mentioned above (Supplementary Information Figure S5). As shown in Figure S5, the majority of the complex remained intact after 48 h (Supplementary Information Figures S5 and S6). The results are in agreement with the UV-Vis experiments, which do not show significant changes between 24 and 48 h (Supplementary Information Figure S4).

### 2.3. Antiproliferative Activity

**Figure 1:**
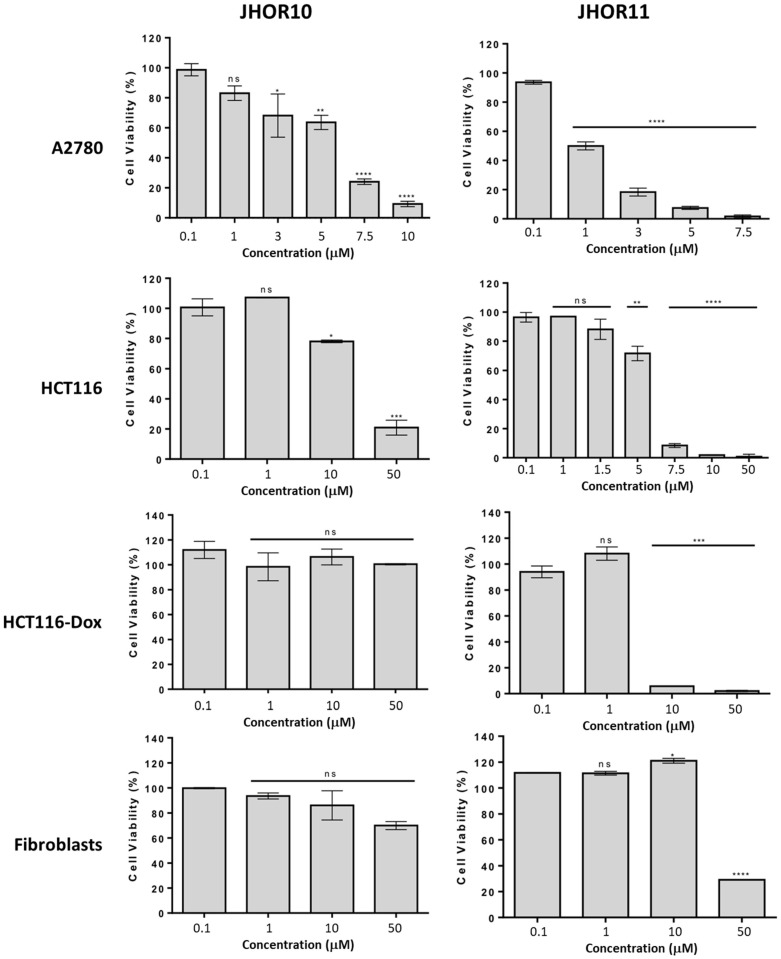
Antiproliferative activity of JHOR10 and JHOR11 after 48 h in A2780, HCT116 and Doxorubicin-resistant HCT116 (HCT116-Dox) cell lines and in normal human dermal fibroblasts. Data were normalized to the control (0.1% (*v*/*v*) DMSO) with bars representing the mean ± SD of at least three independent experiments. * *p* value < 0.05, ** *p* value < 0.01, *** *p* value < 0.005, **** *p* value < 0.0001, n.s. not significant.

The cytotoxic potential of the complexes was evaluated by assessing their viability using the 3-(4,5-dimethylthiazol-2-yl)-5-(3-carboxymethoxyphenyl)-2-(4-sulfophenyl)-2H tetrazolium, inner salt (MTS) colorimetric assay in three tumor cell lines—ovarian carcinoma, A2780, colorectal carcinoma, HCT116, and colorectal carcinoma resistant to doxorubicin, HCT116-Dox and in normal human primary dermal fibroblasts (Figure 1 and Figure S7). The calculation of the relative IC_50_ revealed that both complexes presented higher antiproliferative effects in A2780, with JHOR11 showing lower IC_50_ than cisplatin in this cell line (Table 1, Figure 1). While JHOR10 had an IC_50_ higher than 50 μM in HCT116-Dox, whereas JHOR11 had an IC_50_ in Dox-resistant cells (6.8 μM) in the same order of magnitude of the IC_50_ of the sensitive cell line (5.5 μM) (Table 1). These are interesting results since they suggest that alteration of the counter anion is enough to surpass the MDR in this cell line. The differences in cytotoxicity after alterations of the counter anion were previously described by Zhang et al. (2018) [21], who compared the effect of the counter anion in an iridium(III) complex, and observed pronounced differences in cell cycle, apoptosis and ROS production when using complexes containing small, such as OTf, or bulk counter anions, such as BPh_4_. The different cytotoxic effect caused by the two compounds is also observed in normal cells, with JHOR10 presenting an IC_50_ higher than 50 μM and JHOR11 with an IC_50_ of 44.5 μM in fibroblasts (Figure 1, Table 1). Nevertheless, the selective index of the complexes (37.4 for JHOR11 in A2780 and 8.1 in HCT116), together with the higher cytotoxicity of JHOR11 compared to cisplatin (Table 1, Figure 1 and Supplementary Information Figure S7), make these complexes promising for further biological studies. Due to the antiproliferative effect of JHOR11 on A2780, this cell line was selected to evaluate the mechanisms of cytotoxicity of the complex.

### 2.4. Complexes Internalization in Cells

**Figure 2:**
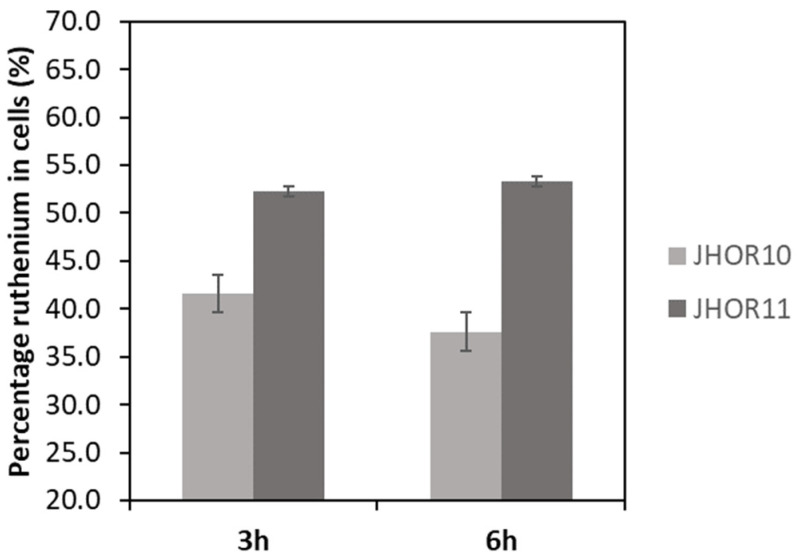
Internalization of JHOR10 and JHOR11 in A2780 cell line. Cells were incubated for 3 h and 6 h in the presence of 10 µM of each complex and the percentage of Ru within cells analyzed by ICP-AES.

The internalization of both complexes by A2780 cells was then examined to understand if the counter anions can influence a different uptake of the complex and internalization, and in that regards explain the discrepancy between the observed IC_50_ for each complex (Figure 1, Table 1). Cells were exposed for 3 h and 6 h to 10 μM of each complex and the percentage of ruthenium inside cells was quantified via inductively coupled plasma—atomic emission (ICP-AES). The analysis of the results presented in Figure 2 showed that there is a higher internalization of JHOR11 relative to JHOR10, that tends to increase throughout time in the first complex, and to decrease in the second complex. These results suggest that, as postulated, counter anions might influence the uptake rate of the complex by cells, which result in a higher cytotoxicity for JHOR11 (Figure 1 and Figure 2 and Table 1).

Previously, other authors have already described the effect of the counter anion on the in vitro cytotoxicity exhibited by the Ruthenium arene complexes, which was found to also be dependent on the anionic counterion [24]. In our study, we show that the counter anion affects the complexes’ internalization rate. However, we also demonstrate, that once inside the cells in the complex in particular, the presence of the phendion ligand dictates their mechanism of action as previously described [15,16].

Quesada and collaborators previously described the structural effect of these counterions on the metal cations chemical properties [25]. Indeed, the asymmetric nature of the CF_3_SO_3_^−^ ions (in contrast to PF_6_^−^) might make JHOR10 uptake by cells difficult.

### 2.5. Mechanisms of Cytotoxicity

**Figure 3:**
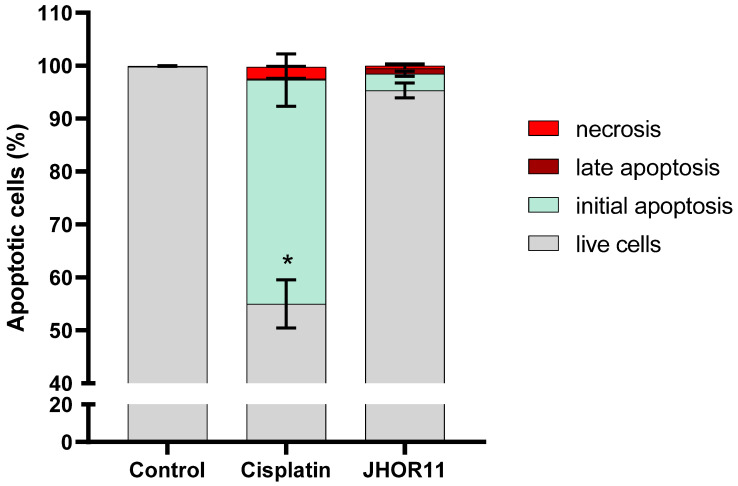
Percentage of apoptotic A2780 cells after 48 h exposure to IC_50_ concentration of JHOR11 evaluated by flow cytometry. DMSO 0.1% (*v*/*v*) was the vehicle control and cisplatin 3.5 μM was used as positive control. Bars represent the mean ± SD of at least three independent experiments. * *p* value < 0.05.

To understand if the loss of cell viability of an A2780 cell incubated with JHOR11 is due to cell apoptosis, cells were incubated with the IC_50_ of the complex and then double-stained with Annexin V, conjugated with AlexaFluor 488 and with propidium iodide (PI) to discriminate between live cells (unstained), cells in initial apoptosis (green-stained cells), cells in late apoptosis (double-stained cells) and cells in necrosis (red-stained cells). The flow cytometry analysis revealed that only 3% of cells were apoptotic after a 48 h exposure to the IC_50_ of the complex (Figure 3).

**Figure 4:**
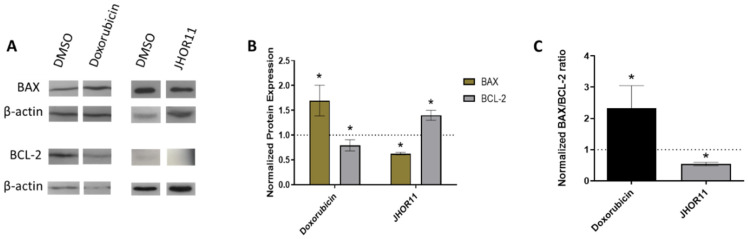
Quantification of apoptotic index of A2780 cells. (**A**) Western blot for quantification of BAX, BCL-2 and β-actin in A2780 cells after 48 h incubation with IC_50_ of JHOR11, 0.1 μM Doxorubicin as positive control or 0.1% (*v*/*v*) DMSO (control). (**B**) BAX and BCL-2 protein expression after normalization to respective β-actin and control. The value of the control is represented as a dotted line. (**C**) Apoptotic index calculated by BAX/BCL-2 ratio. The value of the control is 1, represented as a dotted line. Bars represent the average ± SD of three independent experiments. * *p* value < 0.05 relative to control.

The intrinsic or mitochondrial apoptotic pathway is triggered by signals such as DNA damage or deprivation of growth factors and is regulated by anti-apoptotic BCL-2 family proteins [26,27,28]. When cells are healthy, BCL-2 interacts with the cytoplasmic monomer form in the pro-apoptotic BAX protein [26,27,28]. When the intrinsic apoptosis is induced, BAX is incorporated into the mitochondrial outer membrane as a homo-oligomer, resulting in the dissipation of the mitochondrial membrane potential (ΔΨm) [26,27,28]. The ratio between BAX and BCL-2 protein expression indicates the level of susceptibility of the cell to the intrinsic apoptotic pathway [26,27,28]. The BAX and BCL-2 protein expression was quantified by Western blot analysis to calculate the apoptotic index of cells after 48 h incubation with IC_50_ of JHOR11, using the quantification of β-actin as the internal control and 0.1% (*v*/*v*) DMSO as the vehicle control (Figure 4). Results showed that the apoptotic index result of A2780 cells exposed to JHOR11 is lower than 1 (Figure 4C), confirming the previous apoptotic data (Figure 3) and suggesting that the complex does not induce intrinsic apoptosis.

**Figure 5:**
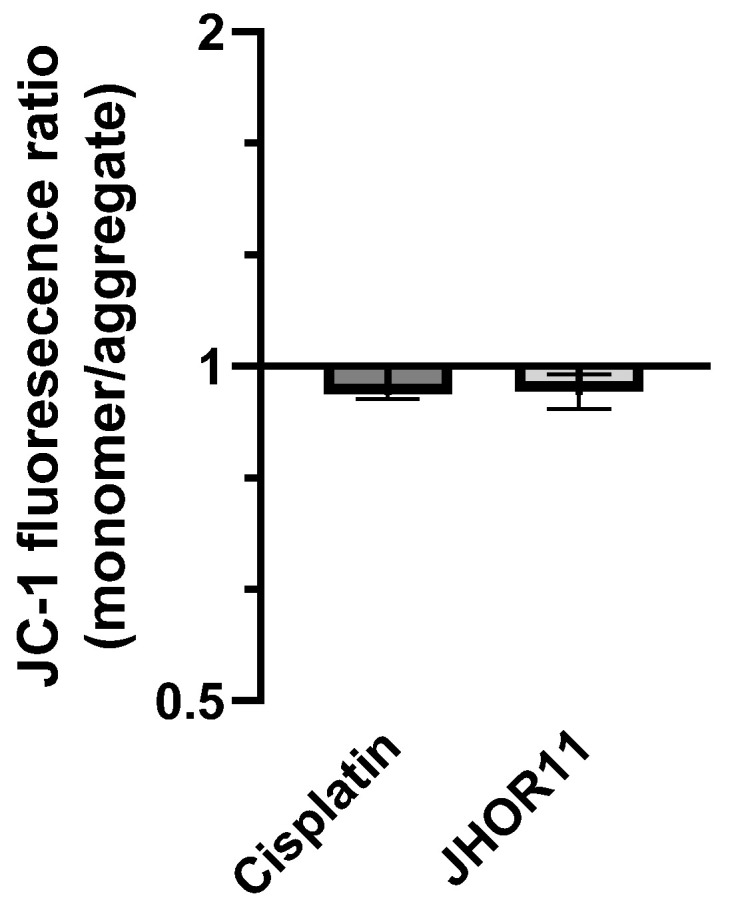
Mitochondrial potential evaluated by the JC-1 monomer/aggregate fluorescence ratio after 48 h incubation of A2780 cells with IC_50_ of JHOR11. DMSO (0.1% (*v*/*v*)) was the vehicle control and cisplatin 3.5 µM was used as the positive control. Data were normalized to the vehicle control, and bars represent the mean ± SD of at least three independent experiments.

Moreover, to further confirm that JHOR11 does not induce intrinsic apoptosis, the ΔΨm was examined using the lipophilic cation dye JC-1. This dye accumulates in the mitochondria of cells, presenting red fluorescence, characteristic of the aggregated form (JC-1 aggregated), in healthy cells, and green fluorescence, characteristic of monomer form (JC-1 monomer), when the mitochondrial membrane is compromised [29]. Hence, the ΔΨm of the cell might be evaluated through the JC-1 monomer/aggregate fluorescence ratio, resulting in a ratio higher than 1 in cells with compromised mitochondrial membrane [29]. Figure 5 shows that after 48 h of exposure of A2780 cells to the JHOR11 complex, a decrease in the monomer/aggregate fluorescence ratio (but with no statistical significance) was observed, which goes in line with the above results from Figure 3 and Figure 4 and the non-induction of the intrinsic apoptosis pathway.

**Figure 6:**
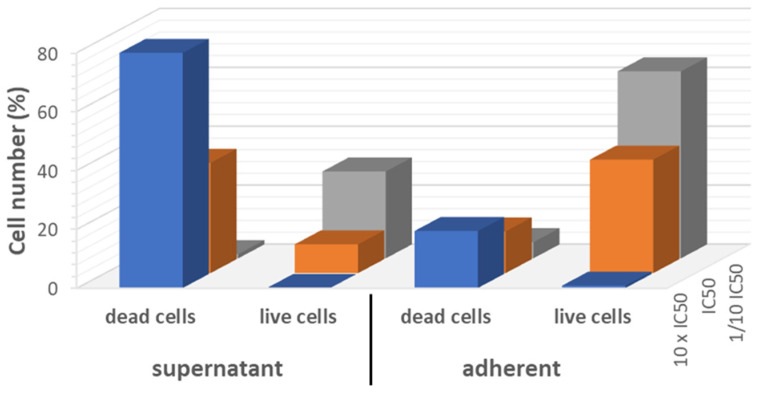
Percentage of live and dead A2780 cells in the supernatant or adherent after 48 h of exposure to the IC_50_ of JHOR11, or to a concentration 10 times higher than the IC_50_ of JHOR11 (10 × IC_50_), or to a concentration 10 times lower than the IC_50_ of JHOR11 (1/10 IC_50_).

To clarify the antiproliferative activity of the JHOR11 complex, the cell death of A2780 was analyzed by the Trypan blue exclusion method, which consists of the incubation of cells with Trypan blue dye that only enter cells with compromised membrane; hence, blue cells represent non-viable (dead) cells [30]. A2780 cells were exposed for 48 h to the IC_50_ of JHOR11, and to a concentration 10 times higher and 10 times lower the IC_50_, and the live (transparent) and dead (blue) cells that remained adherent or in the supernatant were counted (Figure 6). A2780 cells were also incubated with 0.1% (*v*/*v*) DMSO as a vector control, and 0.1 µM doxorubicin as positive control (Supplementary Information, Figure S8). A correlation is found between MTS results and Trypan blue (Figure 1 and Figure 6). Interestingly, an increased concentration of adherent cells with compromised membrane is not observed with increasing concentrations of the complex, but there is an increased percentage of cells in the supernatant as the concentration of the complex increases (Figure 6). Moreover, when cells were incubated with 1/10 IC_50_, cells in the supernatant were exclusively live cells (Figure 6). These results suggest that the complex may induce detachment of cells, and the loss of viability observed in the MTS analysis (Figure 1) may be correlated with the detachment of cells.

**Figure 7:**
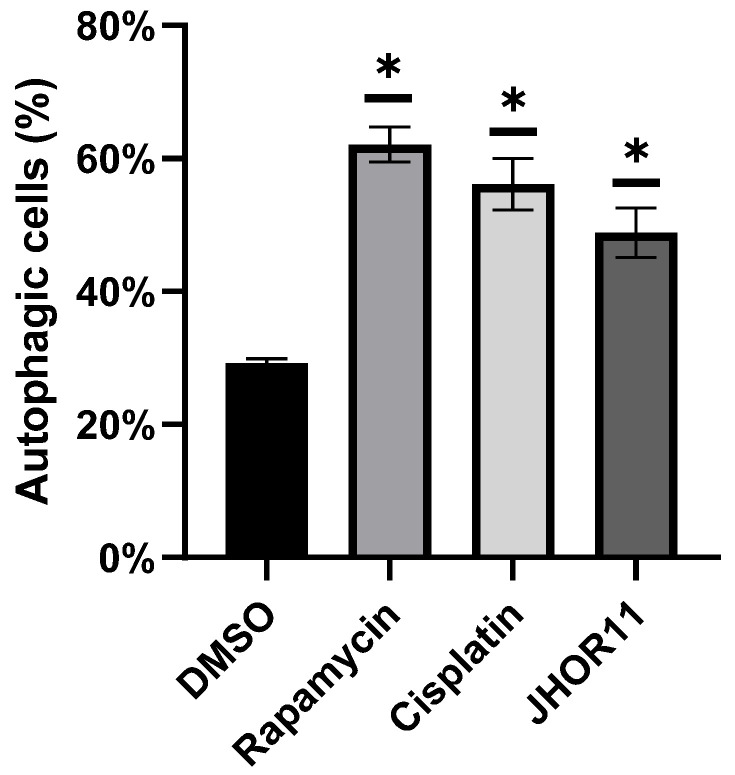
Percentage of autophagic cells after 48 h exposure of A2780 cells to IC_50_ concentration of JHOR11, 0.1% (*v*/*v*) DMSO (vehicle control), 3.5 μM cisplatin and 1.5 μM rapamycin (positive controls). Bars represent the average ± SD of at least three independent experiments. * *p* value < 0.05 relative to vehicle control.

To understand if the cytotoxic effect of the complex is correlated with autophagy, A2780 cells were exposed for 48 h to the IC_50_ of complex JHOR11. They were then incubated with an autophagic fluorescent dye (Abcam) that becomes highly fluorescent when incorporated into pre-autophagosomes, autophagosomes or autophagolysosomes [31]. Cells were also incubated with DMSO 0.1% (*v*/*v*) for vehicle control, and with 1.5 μM rapamycin and 3.5 μM cisplatin as positive controls. After fluorescence quantification by flow cytometry, it was possible to determine that 48.8 ± 7.5% cells presented autophagic events (Figure 7), suggesting that the complex induces an autophagic cell death mechanism.

**Figure 8:**
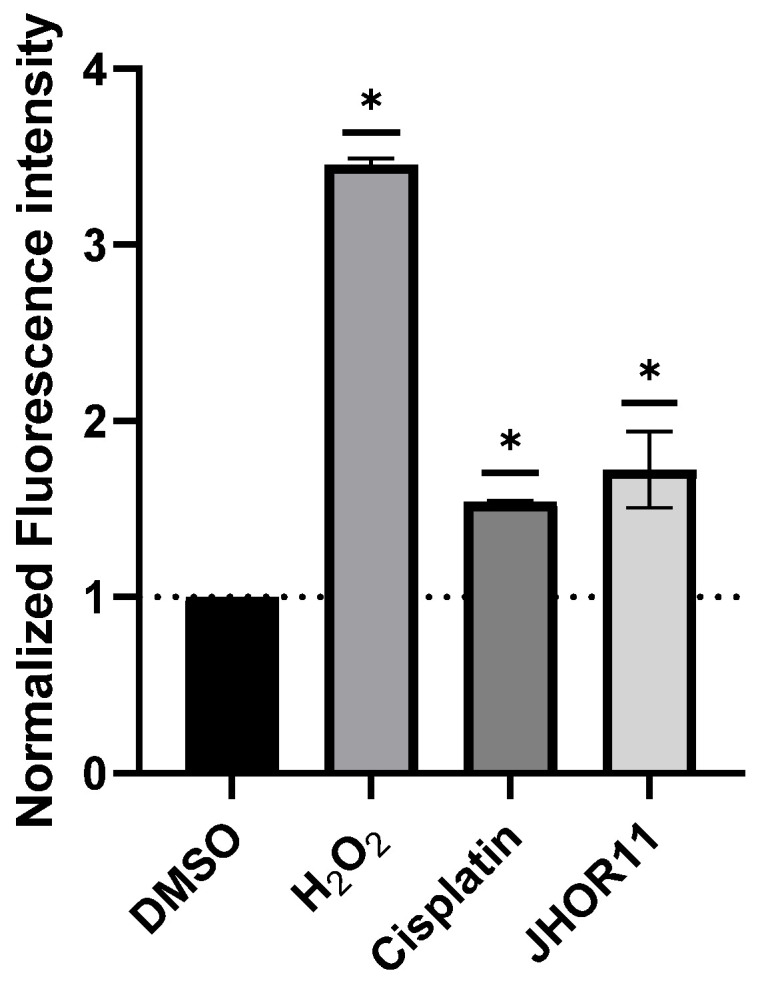
Production of reactive oxygen species (ROS) in A2780 after 48 h exposure to IC_50_ of complex JHOR11, 0.1% (*v*/*v*) DMSO for vehicle control, 25 μM H_2_O_2_ and 3.5 μM cisplatin as positive controls. Bars represent the average ± SD of at least three independent experiments of the fluorescence intensity normalized to fluorescence intensity of the vector control, represented as a dotted line. * *p* value < 0.05.

The triggering of autophagy by RuII(*p*-cymene) complexes was previously described [18,32], suggesting that this is a common mechanism of the cytotoxicity of Ru complexes. Since the cytotoxicity of Ru(II) complexes is commonly accompanied by the induction of reactive oxygen species (ROS) [18,33], the ROS concentration was quantified in A2780 cells exposed for 48 h to IC_50_ concentration of JHOR11, using DMSO 0.1% (*v*/*v*) as a vehicle control, and 3.5 µM cisplatin and 25 µM hydrogen peroxide (H_2_O_2_) as positive controls. The incubation with the dye 2′,7′-dichlorodihydrofluorescein diacetate (H_2_DCF-DA) that, in the presence of peroxides, emits green fluorescence after oxidation to 2′,7′-dichlorofluorescein (DCF) [31], followed by fluorescence quantification by flow cytometry, allowed us to infer the induction of ROS. Results suggest that 1.72 ± 0.4 times increase in intracellular ROS in cells incubated with complexes, relative to cells incubated with vector control (Figure 8), suggesting that complex JHOR11 induces oxidative stress in A2780 cells.

### 2.6. Cytostatic Effect

**Figure 9:**
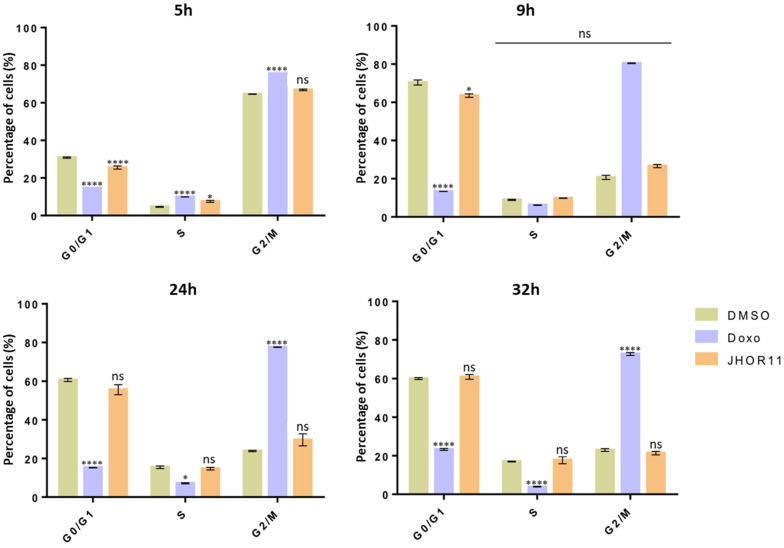
Analysis of the cell cycle for 5 h, 9 h, 24 h or 32 h of A2780 cells after exposure to IC_50_ of complex JHOR11, 0.1% (*v*/*v*) DMSO as vehicle control or 0.1 µM doxorubicin (Doxo) as positive control. Bars represent the average ± SD of at least three independent experiments. * *p* value < 0.05, **** *p* value < 0.001, n.s.—not significant relative to respective vehicle control sample.

To understand if the complex also presents a cytostatic effect, the cell cycle of A2780 cells was analyzed by DNA staining with PI, after double synchronization with thymidine at the S phase. After DNA quantification by flow cytometry, it was possible to verify that complex JHOR11 induces a slight increase in cells in G2/M phase at 24 on those normalized at 32 h, relative to cells incubated with the vehicle control (DMSO) (Figure 9). On the contrary, as expected, doxorubicin induced a cell cycle arrest at G2/M phase (Figure 9).

**Figure 10:**
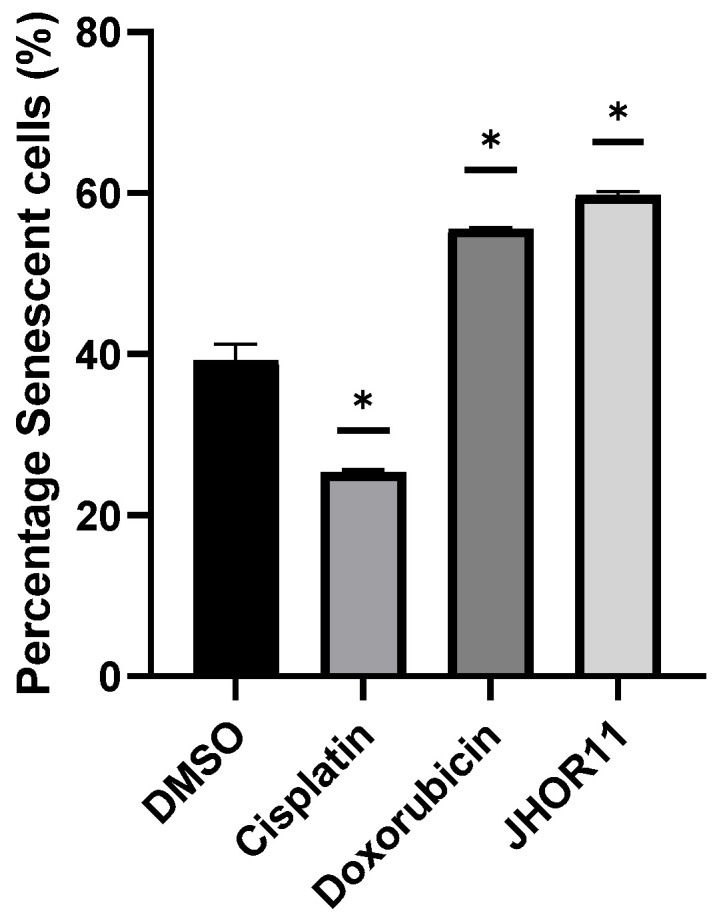
Quantification of senescent A2780 cells after 48 h incubation with IC_50_ of complex JHOR11, 0.1% (*v*/*v*) DMSO as vehicle control, 3.5 μM cisplatin and 0.1 μM doxorubicin as positive control. Bars represent the average ± SD. * *p* value < 0.05 relative to control.

The senescence of A2780 cells after 48 h incubation with IC_50_ of JHOR11 was also analyzed to add more insights into the mechanism of action of the complex. Premature cellular senescence is an irreversible state of cell cycle arrest that occurs in response to intrinsic and extrinsic insults to the cell that induce DNA damage, oxidative stress, or nutrient depletion [34]. The percentage of senescent cells was measured using the senescence assay kit (Abcam) that allows the quantification of the senescence-associated expression of beta-galactosidase activity by flow cytometry [35]. The results suggest an increased percentage of senescent cells after incubation with complex JHOR11, relative to when cells are incubated with DMSO, the vector control (Figure 10). The induction of senescence by RuII(*p*-cymene) complexes was previously described by Berndsen and coworkers, that results suggested senescence in A2780 cells and A2780 cells resistant to cisplatin, after observing the formation of chromosome bridges induced by incubation with erlotinib and [Ru(η^6^-*p*-cymene)Cl_2_(1,3,5-triaza-7-phosphaadamantane)] [36]. The results suggest that the main mechanisms induced by JHOR11 that confer antiproliferative ability to the complex, which are correlated with autophagy (Figure 7) and premature cellular senescence (Figure 10), could possibly be due to increased ROS production (Figure 8).

### 2.7. Tumorigenicity

**Figure 11:**
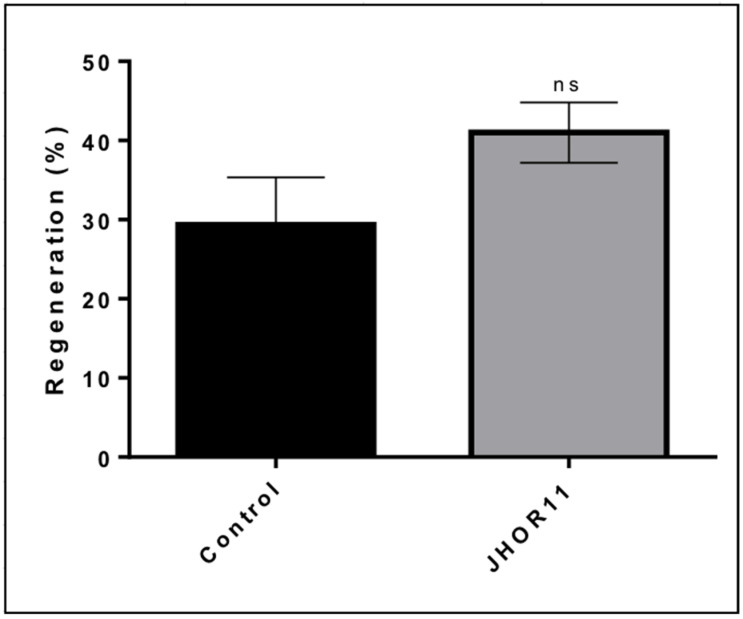
Percentage of wound scratch closure after 24 h incubation with IC_50_ JHOR11 and 0.1% (*v*/*v*) DMSO, as a vector control. Bars represent the mean ± SEM of two independent experiments. n.s.—*p* value not significant.

Cell migration is an essential process for tumor progression, being one of the major events that leads to cancer metastasis [37]. To determine if JHOR11 inhibits cell tumorigenicity, the cell migration ability was analyzed by the wound scratch assay. Hence, a scratch was made with a pipette tip in a monolayer of fibroblasts that were incubated for 24 h with the IC_50_ of the complex. The measure of the wound scratch after 0 h and 24 h allows the determination of the migration ability of the cells. Results suggest that the percentage of regeneration of the wound scratch of cells incubated with the complex has no significant differences with the wound scratch of cells incubated with the vector control (Figure 11), suggesting that the JHOR11 complex does not induce any alteration to the migration ability of the cells.

**Figure 12:**
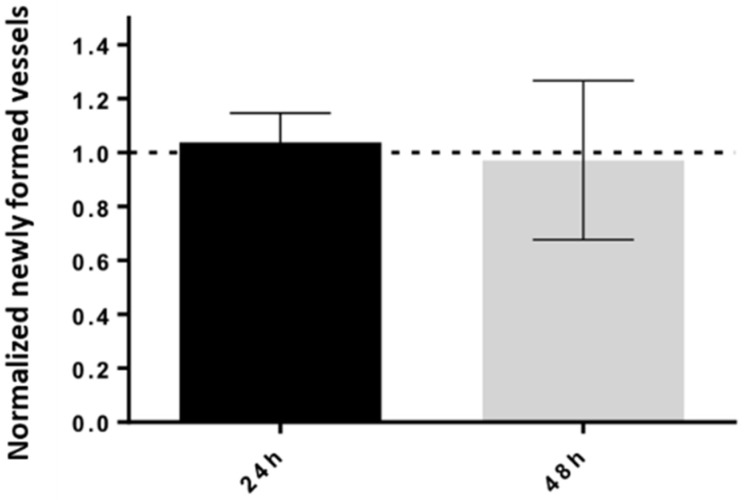
Newly formed vessels after 24 h and 48 h incubation with IC_50_ of JHOR11. Values are normalized to the number of vessels at 0 h and obtained after incubation with 0.1% (*v*/*v*) DMSO at the respective time point in the same embryo. The dotted line at 1.0 represents the value of the DMSO sample normalized to the respective number of vessels at a 0 h time point. Bars represent the average ± SD of six chicken embryos (biological independent assays).

The formation of new vessels, or angiogenesis, in the tumor is preponderant for cancer progression, since it allows to distribute oxygen and nutrients to hypoxic regions, as well as the promotion of metastases, which enables transport of cancer stem cells to other anatomical regions [38]. The anti-angiogenic potential of JHOR11 complex was evaluated using an ex-ovo chorioallantoic membrane (CAM) assay [18,39]. Each chicken embryo was exposed to the IC_50_ of JHOR11 and 0.1% (*v*/*v*) DMSO as vector control and the number of vessels in each treated region was counted at 0 h, and after 24 h and 48 h incubation at 37 °C, as previously described [39]. The newly formed vessels in JHOR11-treated regions were similar to the newly formed vessels in DMSO-treated regions (Figure 12), suggesting that the complex has no anti- or pro-angiogenic effects. Importantly, the complex did not induce the death of the six embryos tested during 48 h, suggesting that it has no major in vivo toxicity.

### 2.8. In Vivo Toxicity Assessment and Xenograft Using Zebrafish Embryos

**Figure 13:**
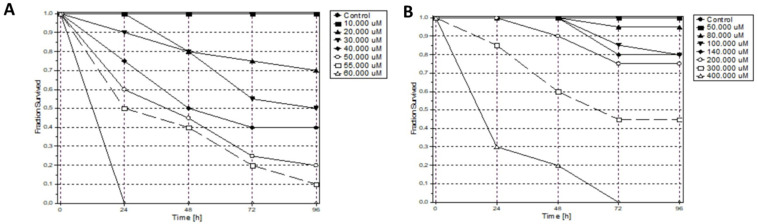
Zebrafish survival in presence of JHOR10 and JHOR11. (**A**) Zebrafish embryo survival when the JHOR10 compound was dissolved in the water. (**B**) Zebrafish embryo survival when the JHOR11 compound was dissolved in the water.

**Figure 14:**
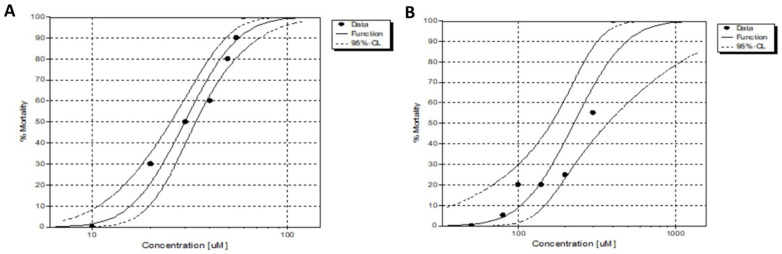
Mortality concentration–effect curve. (**A**) Concentration–effect curve showing the influence of JHOR10 on the mortality of the zebrafish observed after 96 h. (**B**) Concentration–effect curve showing the influence of JHOR11 on the mortality of the zebrafish observed after 96 h.

The toxicity of the JHOR10 and JHOR11 compounds was assayed in zebrafish embryos. There was a NOEC (No Observed Effect Concentration) in the case of JHOR11 of 80 µM (Table 2). The NOEC is the concentration used for the xenotransplantation experiments in order not to affect the normal development of the embryos and being able, in parallel, to assay the effect of the compound over the injected cells. Apart from that, the mortality assayed in the toxicity assays supports the concentration obtained in the NOEC, with a low mortality of the embryos when they are incubated with JHOR11 (Figure 13 and Figure 14).

HCT116 human colorectal cancer cell line was incubated in standard conditions (37 °C and 5% CO_2_) until a confluence of 80% was reached. Afterwards, the cells were concentrated and labelled with DiI and injected into the circulation (Duct of Cuvier) of 48 hpf zebrafish embryos to assay the proliferation in vivo of the cells exposed to JHOR11 (80 μM). JHOR11 was dissolved in the embryo water at 1 dpi (days post-injection) for the embryos to recover from the injection and the establishment of the cancer cells in the caudal hematopoietic tissue (CHT), and was maintained until the end of the experiment at 4 dpi. Imaging of the embryos was performed at 1 dpi and 4 dpi to quantify the fluorescence and area of the cells in the tail of the embryos, where cells invade and grow, and measure their proliferation over time to assess the inhibitory effect of JHOR11 over the cells.

**Figure 15:**
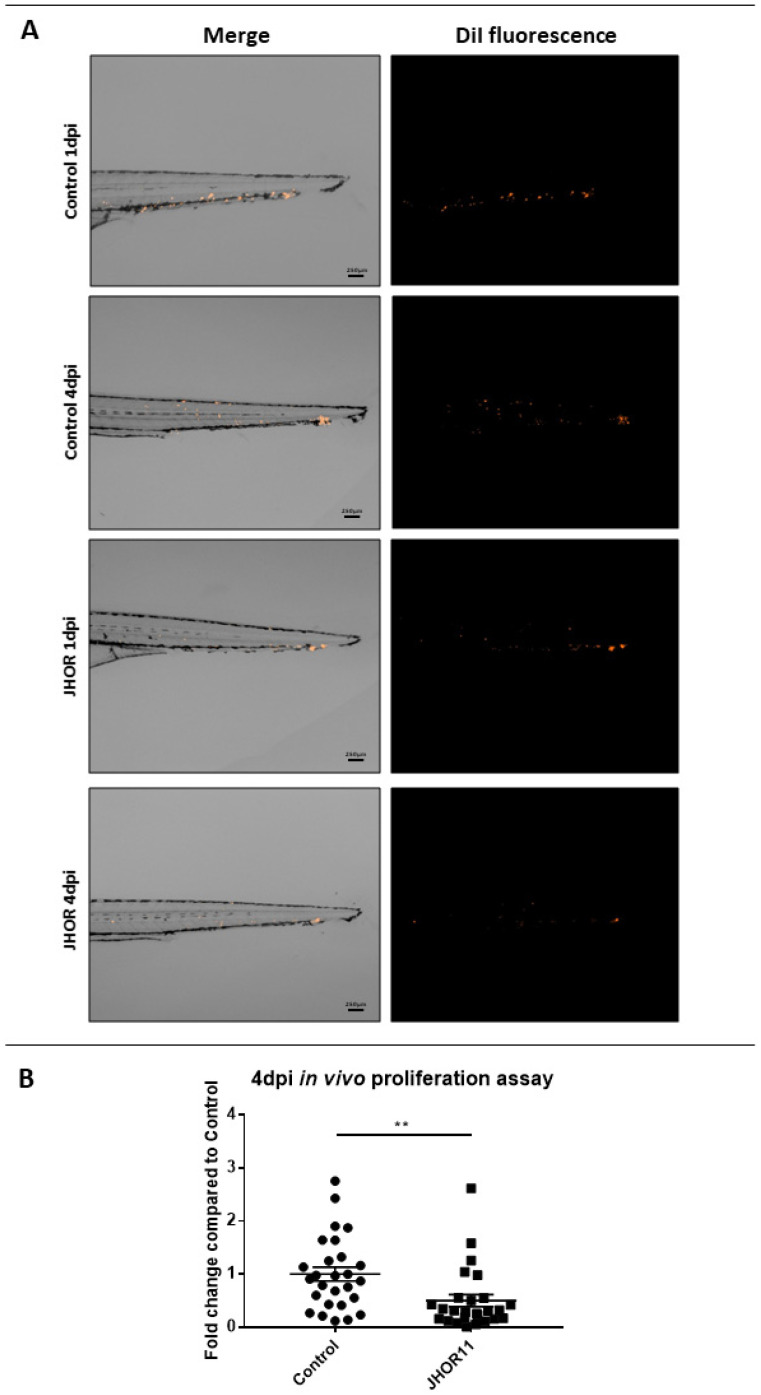
In vivo xenograft assays of human HCT116 colorectal cancer cell line with the JHOR11 (JHOR) treatment. (**A**) Representative images of the tail region of the zebrafish embryos (caudal hematopoietic tissue—CHT) where the DiI HCT116 labelled cells metastasize and proliferate at 1 dpi and 4 dpi for comparison. Main images are a superposition of a fluorescence image and a bright field image of the same embryo. Fluorescence images shows only the labelled cells of the main image. Scale = 250 µm. (**B**) Proliferation analysis based on the images taken at 4 dpi compared with the ones at 1 dpi, representing the fold change normalized to the control condition (n_total_ = 25 embryos/condition, 3 replicates). (** *p* value = 0.062).

The results obtained clearly indicate that JHOR11 affects the HCT116 cells injected in the circulation of the zebrafish embryos, decreasing the proliferation by 45% (* *p* < 0.01) at 4 dpi compared to the control condition (Figure 15).

## 3. Materials and Methods

### 3.1. Synthesis

The synthesis of the metal complexes was carried out under a nitrogen atmosphere using Schlenk techniques. All solvents used were dried prior to use by standard methods. Reagents were purchased from commercial suppliers and used without further purification. Complexes JHOR10 and JHOR11 were prepared following the procedure described in the literature [22] by a reaction of the precursor [Ru(*p*-cymene)Cl_2_]_2_ with the halide abstractor silver triflate for JHOR10 and hexafluorofostate for JHOR11 [22], and next with the phendione ligand.

#### Preparation [Ru{p-C_6_H_4_(Me)(iPr)}{(Dione)Cl][CF_3_SO_3_] (JHOR10)

To a solution of [Ru(*p*-cymene)(Cl)(μ-Cl)]_2_ (0.05 g, 0.0816 mmol) in dichloromethane (20 mL), AgCF_3_SO_3_ (0.04195 g, 0.1632 mmol) was added, and the mixture was refluxed for 16 h. Then, phendione (0.034 g, 0.1632 mmol) was added, and the mixture was further stirred at room temperature for 16 h. The resulting solution was filtered twice through celite to remove the silver chloride which had formed. The filtrate solution was concentrated to ca. 10 mL and diethyl ether was added. The precipitate formed was isolated by filtration, washed with diethyl ether, and dried under vacuum conditions to give JHOR10 as a pure compound. Yield: 74 mg (72%). Elem. Anal. Calcd. for C_23_H_20_ClF_3_N_2_O_5_RuS: C, 43.85; H, 3.20; N, 4.45; S, 5.09. Found: C, 44.07; H, 2.91.; N, 4.12; S, 5.00 IR (ν_max_/cm^−1^): 1255, 1224, 1159, 1029; ν(CF_3_SO_3_). ^1^H NMR (400 MHz, CD_3_CN): δ = 9.56 (d, *J* = 5.6 Hz 2H, H_1,1′_), 8.68 (d, *J* = 7.9 Hz, 2H, H_3,3′_), 7.96 (dd, *J* = 5.6 Hz, *J′* = 8.0 Hz 2H, H_2,2′_), 6.07 (d, *J* = 6.3 Hz, 2H, H_8,8′,9,9′_), 5.87 (d, *J* = 6.2 Hz, 2H, H_8,8′,9,9′_), 2.74 (m, 1H, H_11_), 2.27 (s, 3H, H_6_), 1.11 (d, J = 6.9 Hz, H_12,12′_). ^13^C NMR (400 MHz, CD_2_Cl_2_,): δ = 174.97 (C_5,5′_), 160.06 (C_1,1′,2,2′,3,3′_), 154.33 (C_4,4′,13,13′_), 138.73 (C_1,1′,2,2′,3,3′_), 130.76 (C_4,4′,13,13′_), 129.51, (C_1,1′,2,2′,3,3′_), 106.84, (C_7,10_), 104.50, (C_7,10_), 86.63 (C_8,8′,9,9′_), 84.96 (C_8,8′,9,9′_), 31.43 (C_11_), 21.82 (C_12,12′_), 18.63 (C_6_). HRMS (m/z) calcd. for C_22_H_20_ClN_2_O_2_Ru: 481.0251. Found: 481.0250.

### 3.2. Complexes Stability

The stability of complexes was evaluated in DMEM (Dulbecco’s modified Eagle’s medium). Powdered complexes JHOR10 and JHOR11 were solubilized in DMSO and then immediately diluted in DMEM without phenol red (Thermo Fisher Scientific, Waltham, MA, USA) to a final concentration of 100 µM, assuring that the final concentration of DMSO was not higher than 0.1% (*v*/*v*). The UV-visible spectrum was measured after an incubation at 37 °C for 24 or 48 h (DMEM) in a wavelength range between 260 and 800 nm, using a quartz cuvette with 1 cm path length. A total of 2 mg of each compound was dissolved in DMSOd_6_-D_2_O 3:7 (*v*/*v*) 110 mM chloride concentration (NaCl), and the ^1^H NMR were recorded at 0 h and 48 h on a Bruker Avance III 400 MHz.

### 3.3. Cell Culture

Normal dermal human fibroblasts, colorectal carcinoma cell line HCT116 and ovarian carcinoma cell line A2780 were acquired from ATCC (Manassas, VA, USA). HCT116 cell lines resistant to doxorubicin (HCT116-Dox) were derived from the HCT116 cell line, as previously described [18]. Fibroblasts and HCT116 cells were cultured in Dulbecco’s modified Eagle medium (DMEM, Thermo Fisher Scientific) and A2780 cell line was cultured on Roswell Park Memorial institute 1640 (RPMI-1640, Thermo Fisher Scientific). Both media were supplemented with 10% (*v*/*v*) fetal bovine serum (FBS, Thermo Fisher Scientific) and a mixture of 100 U/mL penicillin and 100 mg/mL streptomycin. The supplemented media are referred to from this point on as DMEM or RPMI for simplicity. HCT116-Dox was cultured in DMEM supplemented with 3.6 µM doxorubicin (Sigma Aldrich, St. Louis, MO, USA) to maintain resistance. All experiments underwent at 37 °C, 5% (*v*/*v*) CO_2_ and 99% (*v*/*v*) humidity in the dark.

### 3.4. Complexes Internalization

To infer the complexes’ internalization, A2780 cells were seeded in 25 cm^2^ flasks at a density of 1 × 10^6^ cells/flask and let to adhere for 24 h at 37 °C, 5% (*v*/*v*) CO_2_, 99% (*v*/*v*) humidity. The medium was replaced by a fresh medium supplemented with 10 μM of each complex (to guarantee a concentration high enough for detection) and incubated for 3 h and 6 h. The supernatant was then collected, and cells were detached and pelleted at 500× *g* during 5 min. Aqua regia, consisting of a 1:3 mixture of HNO_3_ and HCl, was added to each supernatant and pellet, and the ruthenium in each sample was quantified in a Horiba Jobin Yvon inductively coupled plasma atomic emission spectrometer as a paid service (Analytical Laboratory, Department of Chemistry, FCT-NOVA).

### 3.5. Antiproliferative Activity

Cells were seeded in a 96-well plate in a density of 7500 cells/well, and after 24 h was submitted to a concentration range between 0.1–50 µM of complexes JHOR10 and JHOR11. DMSO with a 0.1% (*v*/*v*) concentration was used as vector control and cisplatin was used as positive control in all replicates. After 48 h incubation, cellular viability was inferred with the Cell Titer 96^®^ Aqueous One solution cell proliferation assay (Promega, Madison, WI, USA) according to the manufacturer’s instructions and procedures previously described [18,40]. The absorbance of the produced Formazan was quantified with a Tecan microplate reader, Infinite M200 (Tecan, Mannedorf, Switzerland) and the analysis of the dose–response curves to determine the relative IC_50_ was performed with GraphPad Prism 8.2.1 software (GraphPad Software, La Jolla, CA, USA).

### 3.6. Evaluation of Apoptosis with Annexin V—Alexa Fluor 488/Propidium Iodide Double Staining

The double staining of the A2780 cells incubated with JHOR11 for apoptosis evaluation followed procedures previously described [17]. Briefly, A2780 cells were seeded on 6-well plates in a density of 2 × 10^5^ cells/well, and incubated for 48 h with IC_50_ JHOR11, 0.1% (*v*/*v*) DMSO as vector control, and 3.5 µM cisplatin as positive control. Cells were then detached from the wells and double stained using the dead cell apoptosis kits with Annexin V for flow cytometry (Thermo Fisher Scientific) according to manufacturer’s instructions. The percentage of live cells (unstained), cells in initial apoptosis (stained with annexin V-Alexa Fluor 488), cells in late apoptosis (double stained with annexin V-Alexa Fluor 488 and propidium iodide) or cells in necrosis (stained with propidium iodide) was quantified using an Attune acoustic focusing flow cytometer and respective software (Thermo Fisher Scientific).

### 3.7. Western Blot to Determine the Apoptotic Index

The apoptotic index was determined by quantification of BAX and BCL-2 protein expression by Western blot, as previously described [18]. Briefly, 4 × 10^6^ cells were seeded on T-flasks with 25 cm^2^ for 24 h and then the medium was replaced by a fresh medium supplemented with IC_50_ JHOR11, 0.1% (*v*/*v*) DMSO (vector control) or 0.1 µM doxorubicin (positive control). After 48 h incubation, cells were detached with a cell scraper and BAX, BCL-2 and β-actin proteins were quantified in a PVDF membrane [18]. The apoptotic ratio BAX/BCL-2 was calculated after normalization of the BAX and BCL-2 band intensity with respective β-actin band intensity and to the values of the DMSO-treated sample, measured using ImageJ2 software [41].

### 3.8. Mitochondrial Membrane Potential

The mitochondrial potential was quantified using the JC-1 mitochondrial membrane potential assay kit (Abnova, Taipé, Taiwan) according to manufacturer’s instructions and as previously described [18]. Briefly, 2 × 10^5^ A2780 cells were seeded on each well of a 6-well plate and incubated for 48 h with IC_50_ JHOR11, 0.1% (*v*/*v*) DMSO or 3.5 µM cisplatin. Cells with red (JC-1 aggregated) and green (JC-1 monomer) fluorescence were quantified with an Attune acoustic focusing flow cytometer and respective software (Thermo Fisher Scientific), and fluorescence ratios normalized to control samples incubated with DMSO.

### 3.9. Trypan Blue Exclusion Method

A2780 cells were seeded on a 24-well culture plate at a density of 37,500 cells/well. After 24 h to assure cell adherence, the medium was replaced by fresh RPMI supplemented with 0.1% (*v*/*v*) DMSO, 0.1 µM Doxorubicin or JHOR11 complex in three different concentrations: 1/10 IC_50_, IC_50_ or 10 × IC_50_. After 8 h, 24 h or 48 h incubation, the supernatant was removed and mixed with trypan blue (Thermo Fisher Scientific) to a final concentration 0.1% (*w*/*v*). In parallel, cells were detached from the well, centrifuged at 500× *g* and pellet was resuspended in 0.1% (*w*/*v*) Trypan blue. The number of viable cells and cells with compromised membrane (stained with blue) in both fractions were counted in a haemocytometer (Hirschmann, Eberstadt, Germany) and a CXX41 inverted microscope (Olympus, Tokyo, Japan).

### 3.10. Autophagy

The percentage of autophagic cells was evaluated using the Autophagy assay kit (Abcam, Cambridge, UK) according to the manufacturer’s instructions and procedures previously described [18]. Briefly, A2780 cells were seeded on 6-well plates with a density of 2 × 10^5^ cells/well and incubated for 48 h with IC_50_ JHOR11, 0.1% (*v*/*v*) DMSO (vector control), 1.5 µM rapamycin or 3.5 µM cisplatin (positive controls). The fluorescence quantification and percentage of autophagic cells was performed on an Attune acoustic focusing flow cytometer and respective software (Thermo Fisher Scientific).

### 3.11. Production of Reactive Oxygen Species

The production of ROS was evaluated using the dye 2′,7′-dichlorodihydrofluorescein diacetate (H_2_DCF-DA, ThermoFisher Scientific) according to previously described procedures [18]. A2780 cells were seeded on 6-well plates in a cell density of 2 × 10^5^ cells/well and incubated for 48 h with IC_50_ JHOR11, 0.1% (*v*/*v*) DMSO (vector control), 25 µM hydrogen peroxide or 3.5 µM cisplatin (positive controls). The fluorescence was quantified in an Attune acoustic focusing cytometer and respective software (Thermo Fisher Scientific) and normalized to the fluorescence of vector control sample.

### 3.12. Cell Cycle Analysis

The cell cycle analysis was performed by DNA staining with PI according to procedures previously described with few modifications [18,42]. A2780 cells were seeded in 6-well plates in a cell density of 4 × 10^5^ cells/well and the cell cycle was synchronized in the S-phase by double blocking with 2 mM thymidine [18]. After the second block, cells were incubated with fresh medium supplemented with IC_50_ JHOR11, 0.1% (*v*/*v*) DMSO, as vector control or 0.1 µM doxorubicin, that induce cell cycle blocking in the G2/M phase [39]. After 5 h, 9 h, 24 h or 32 h, cells were detached, fixed with 80% (*v*/*v*) ethanol, incubated with 50 mg/mL RNase A (NZYtech, Lisbon, Portugal) and stained with 25 µg/µL PI (Thermo Fisher Scientific). The fluorescence of each sample was analyzed in an Attune acoustic focusing cytometer and respective software (Thermo Fisher Scientific).

### 3.13. Senescence Assay

The cell senescence analysis was performed using the Senescence assay kit (beta galactosidade fluorescence, Abcam, Cambridge, UK) according to the manufacturer’s instruction with few modifications. Briefly, A2780 cells were seeded in 24 well-plates to a final cell density of 4 × 10^5^ cells/well. After 24 h incubation, to assure cell adhesion to the wells, the medium was replaced by fresh media supplemented with 0.1% (*v*/*v*) DMSO, for vector control, 3.5 µM cisplatin, 0.1 µM Doxorubicin, or IC_50_ JHOR11. After 48 h, the medium was removed and cells were incubated with senescence dye in fresh media for 1 h 30 min. Cells were washed twice with wash buffer and collected by trypsinization and centrifugation at 500× *g* for 5 min. Pelleted cells were washed with wash buffer, resuspended in the same buffer and the percentage of fluorescent cells in each sample was immediately measured in an Attune focusing flow cytometer and respective software (Thermo Fisher Scientific).

### 3.14. Wound Scratch Assay

For wound scratch assay normal human dermal fibroblasts were seeded on 35 mm^2^ tissue plates in a cell density of 4 × 10^5^ cells/plate and incubated at 37 °C, 5% (*v*/*v*), 99% (*v*/*v*) relative humidity until reach confluence. A sterile micropipette tip was then used to make a scratch on the confluent monolayer and medium was replaced by fresh medium supplemented with IC_50_ JHOR11 or 0.1% (*v*/*v*) DMSO, as vector control. Three regions of the scratch were imaged after 0 h and 24 h with a digital USB microscope camera (Opti-Tekscope OT-V1, Chandler, AZ, USA) and the size of the wound scratch measured using ImageJ2 software [41].

### 3.15. Ex Ovo CAM Assay

The CAM assay was performed as previously described [18,39,43]. Fertilized eggs with 48 h incubation were opened and stabilized for 24 h in white weighing boats. Two silicone rings were positioned in each embryo and 40 mL IC_50_ JHOR11 in phosphate-buffered saline (PBS) or 0.1% (*v*/*v*) DMSO in PBS, as vector control, were placed in each o-ring. The region delimited by each o-ring was photographed at 0 h, 24 h, 48 h with a digital USB microscope camera (Opti-Tekscope OT-V1). The newly formed veins were counted using ImageJ as previously described [37], and values were presented after normalization with the values obtained for control sample.

### 3.16. Zebrafish Handling and Care

Wild-type zebrafish were housed in 30 L tanks within a water recirculation system under controlled physicochemical conditions: pH 7–7.5, temperature 26 ± 2 °C, conductivity 600 µS/cm and light/dark photoperiod of 14/10 h. The adult fish were fed Gemma Micro 300 (Skretting) twice a day. Fertilized eggs were collected after natural spawning and stored in Petri dishes for 48 h until being injected, or 72 h until being exposed to different concentrations of ruthenium compounds.

All the procedures used in the experiments, fish care and treatment were performed in agreement with the conditions of Animal Care and Use Committee of the University of Santiago de Compostela and the standard protocols of Spain (Directive 2012-63-UE). At the end of the experiments, zebrafish embryos were euthanized by tricaine overdose.

### 3.17. In Vivo Toxicity Assessment Using Zebrafish Embryos

At 72 h post-fertilization (hpf), the embryos were exposed to increasing concentrations of two ruthenium compounds to determine toxicological estimates: LC50 with no observed effect concentration (NOEC) and lowest observed effect concentration (LOEC). The acute toxicity assessment of the compounds was performed following the standards of the OECD Test Guideline Program [44].

The embryos were individually transferred to 96-well plates containing 100 µL of each desired concentration. The plates were then placed in an incubator with a temperature of 26 °C and a photoperiod of 14 h of light and 10 h of darkness. The duration of the experiment was 96 h, and observations were made every 24 h recording the four lethal effects indicated by the OECD: embryo coagulation, absence of somite formation, absence of separation of the yolk tail and absence of heartbeat. All compounds were dissolved with dimethylsulfoxide (DMSO) with a final concentration of 1%. In each test, seven concentrations were analyzed for each compound, these being: JHOR10 (10, 20, 30, 40, 50, 55, 60 µM) and JHOR11 (50, 80, 100, 140, 200, 300, 400 µM).

For the evaluation of toxicity, the mortality data were analyzed by probit analysis with the ToxRat software (ToxRat Solutions. 2003. ToxRat^®^. Software for the statistical analysis of biotests. Alsdorf, Germany) [45].

### 3.18. Cell Culture

The human colorectal carcinoma cell line (HCT116) was cultured as described above (antiproliferative assays).

### 3.19. Zebrafish Embryo Xenograft Assays

Zebrafish embryos were harvested and incubated at 28 °C for the first 48 h. Al 48 hpf, embryos were anesthetized with 0.003% tricaine (Sigma). The human colorectal carcinoma cells with 80% confluence were trypsinized and harvested (approximately one million cells). Cells were labelled with Dil lipophilic dye and concentrated in 10 µL of phosphate-buffered saline (PBS) containing 2% polyvinylpyrrolidone 40 (PVP40) to prevent cell aggregation. They injected 200–300 cells in circulating embryos using a micromanipulador and an electric microijector IM-31 (Narishige). They were used to inject borosilicate glass capillary needles (1 mm O.D. × 0.75 mm I.D.; World Precision Instruments, Sarasota, FL, USA) with an outlet pressure of 34 kPa and an injection time of 30 ms. The embryos were incubated for four days after injection (hpi) al 34 °C in Petri dishes. At 24 hpi the embryos were imaged and the compound JHOR11 was added to the water. The concentration used was 80 µM. For in vivo monitoring of cells within the embryo, images were also taken at 96 hpi.

### 3.20. Zebrafish Embryo Image Analysis

The images were obtained with an AZ-100 Nikon fluorescence stereomicroscope at two different times (day 1 and 3) allowing in vivo monitoring of the proliferating HCT116 cells labelled within the zebrafish embryo. The analysis of the cell progression was carried out using Quantifish Software [46]. This software analyzes each image by measuring its fluorescence above a manually set threshold that produces the integrated intensity of each image as the output. This measure is the result of multiplying the number of positive pixels by the medium intensity of the fluorescence in each image. Integrated intensity was the value further processed to obtain a proliferation ratio and compare the treatment (JHOR11) against the control HCT116 cells without compound added to the embryos (control condition).

### 3.21. Statistical Analysis

Statistical analysis was performed using Graphpad Software (GraphPad Prism version 7.00 for Windows, GraphPad Software, La Jolla, CA, USA, www.graphpad.com (accessed on 22 June 2022)). Graphpad Software was used to perform the statistical analysis of the data performing a comparison using t-test with a confidence interval of 95%.

## 4. Conclusions

In this work, two Ru(II) *p*-cymene 1,10-phenanthroline-5,6-dione with general formulas [(η^6^-arene)Ru(L)Cl][X] where X = CF_3_SO_3_, (JHOR10) and PF_6_ (JHOR11) were prepared and their anticancer activity was evaluated in an ovarian carcinoma cell line (A2780), in a colorectal carcinoma cell line (HCT116) and in a doxorubicin-resistant colorectal carcinoma cell line (HCT116-dox). Interestingly, the alteration of the counter anion resulted in the alteration of the cytotoxicity in the resistant cell line, with JHOR11 showing improved antiproliferative ability relative to JHOR10. Moreover, the difference in the counter anion might induce changes in the uptake of the complexes, with JHOR10 showing a lower internalization rate that might correlate with its lower cytotoxicity. The examination of the mechanisms responsible for the antiproliferative effect of JHOR11 revealed that it was mainly based on the production of ROS that induce autophagy and cellular senescence. Importantly, the complex presented no tumorigenic effect, based on the wound scratch and ex-ovo CAM assays, nor major toxicity in chicken and zebrafish embryos. Moreover, the JHOR11 complex reduces in vivo the proliferation of the HTC116 human colorectal cancer cell line injected in zebrafish embryos. These results show that these are promising compounds for clinical applications, and that there is the possibility of using the JHOR10, with counter anion CF_3_SO_3_, as first-line treatment and JHOR11, with counter anion PF_6_, in MDR tumors.

## Figures and Tables

**Table 1:** Relative IC_50_ (in µM) after 48 h of incubation of complexes JHOR10 and JHOR11 in A2780, HCT116 and HCT116-Dox cell lines and in normal human dermal fibroblasts. n.d.—not determined.

	IC_50_ (μM)			
Complexes	A2780	HCT116	HCT116-Dox	Fibroblasts
JHOR10	5.3 ± 0.3	25.7 ± 1.3	>50	>50
JHOR11	1.2 ± 0.1	5.5 ± 0.3	6.8 ± 0.3	44.5 ± 2.2
Cisplatin	1.9 ± 0.2	15.6 ± 5.3	n.d.	n.d.

**Table 2:** LC_50_ (with 95% confidence limit), NOEC and LOEC for ruthenium compounds JHOR10 and JHOR11.

Compound	LC_50_ (µM)	C.L (95%)	NOEC (µM)	LOEC (µM)
JHOR10	29.83	25.31–33.97	10.00	20.00
JHOR11	225.38	161.86–370.40	80.00	10.00

C.L Confidence limits; NOEC (No Observed Effect Concentration); LOEC (Lowest Observed Effect Concentration).

## Data Availability

Not applicable.

## Supplementary Materials

The supporting information can be downloaded at: https://www.mdpi.com/article/10.3390/ijms232113594/s1.
