# Supplementary material for: In Vitro and In Vivo Biological Activity of Ruthenium 1,10-Phenanthroline-5,6-dione Arene Complexes
Source: Int J Mol Sci. 2022 Nov 6;23(21):13594. doi: 10.3390/ijms232113594 (PMC9656482; doi:10.3390/ijms232113594)
Supplement: Supplementary file 1 [file ijms-23-13594-s001.zip › ijms-1941728-supplementary.pdf]

International Journal of *Molecular Sciences*

MDPI

Article

In Vitro and In Vivo Biological Activity of Ruthenium 1,10-Phenanthroline-5,6-dione Arene Complexes

Oscar A. Lenis-Rojas [1,*], Catarina Roma-Rodrigues [2,3], Beatriz Carvalho [2,3], Pablo Cabezas-Sainz [4], Sabela Fernández Vila [4], Laura Sánchez [4,5], Pedro V. Baptista [2,3], Alexandra R. Fernandes [2,3,*] and Beatriz Royo [1]

1 ITQB NOVA, Instituto de Tecnologia Química e Biológica António Xavier, Universidade Nova de Lisboa, Av. da República, 2780-157 Oeiras, Portugal
2 Associate Laboratory i4HB—Institute for Health and Bioeconomy, NOVA School of Science and Technology, NOVA University Lisbon, 2819-516 Caparica, Portugal
3 UCIBIO—Applied Molecular Biosciences Unit, Department of Life Sciences, NOVA School of Science and Technology, NOVA University Lisbon, 2819-516 Caparica, Portugal
4 Departamento de Zoología Genética y Antropología Física, Facultad de Veterinaria, Universidad de Santiago de Compostela, Campus de Lugo, 27002 Lugo, Spain
5 Preclinical Animal Models Group, Health Research Institute of Santiago de Compostela (IDIS), 15706 Santiago de Compostela, Spain
* Correspondence: oscar.rojas@itqb.unl.pt (O.A.L.-R.); ma.fernandes@fct.unl.pt (A.R.F.)

Supplementary Materials

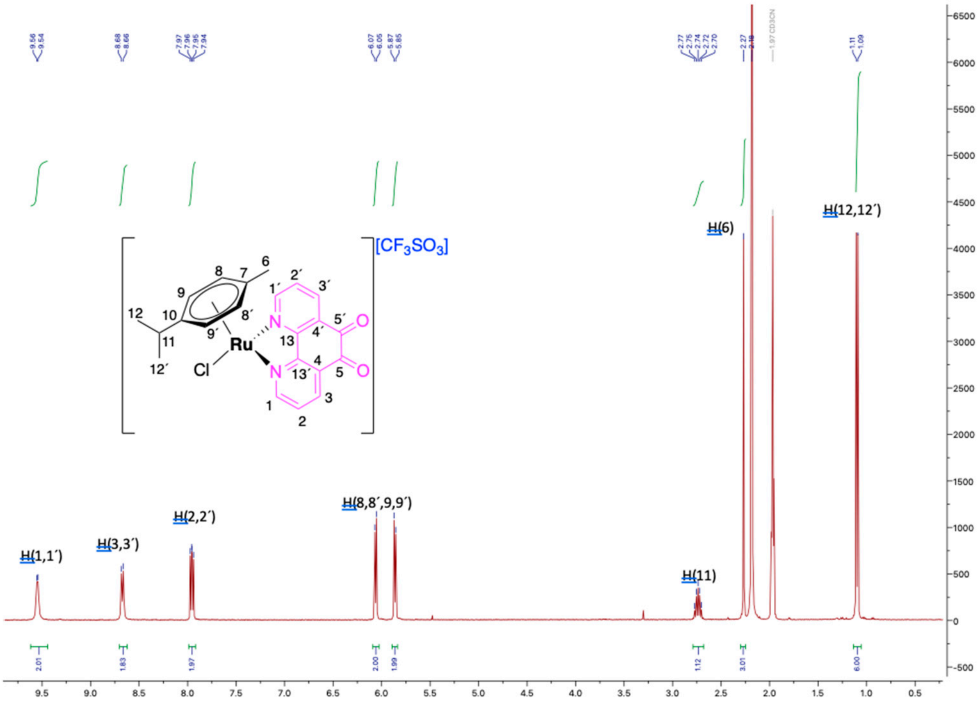

Figure S1. ^{1}H NMR spectra of JHOR10.

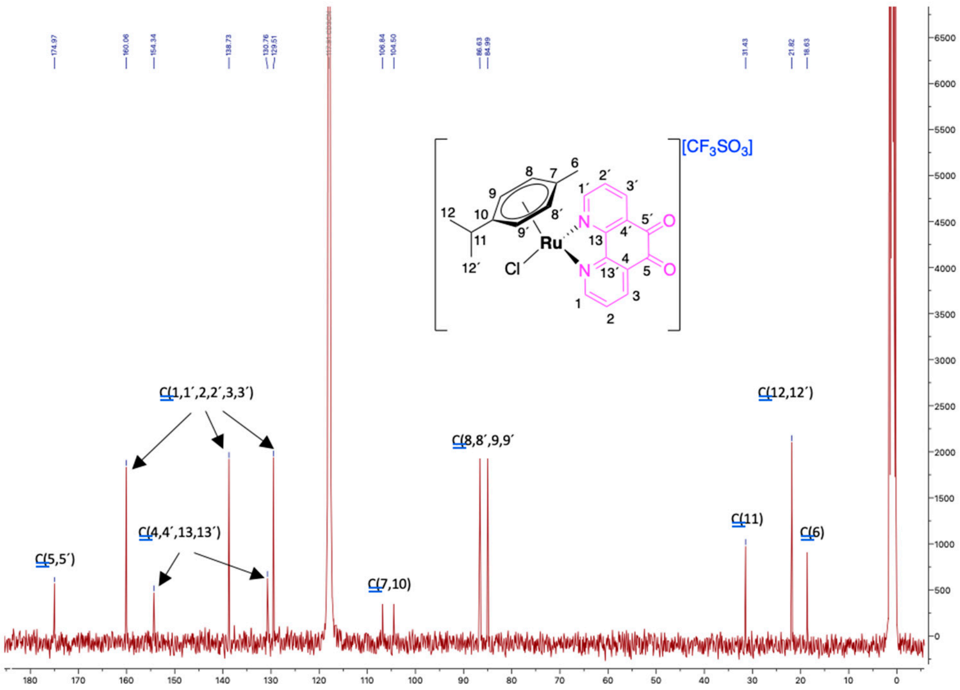

Figure S2. ^{13}C NMR spectra of JHOR10.

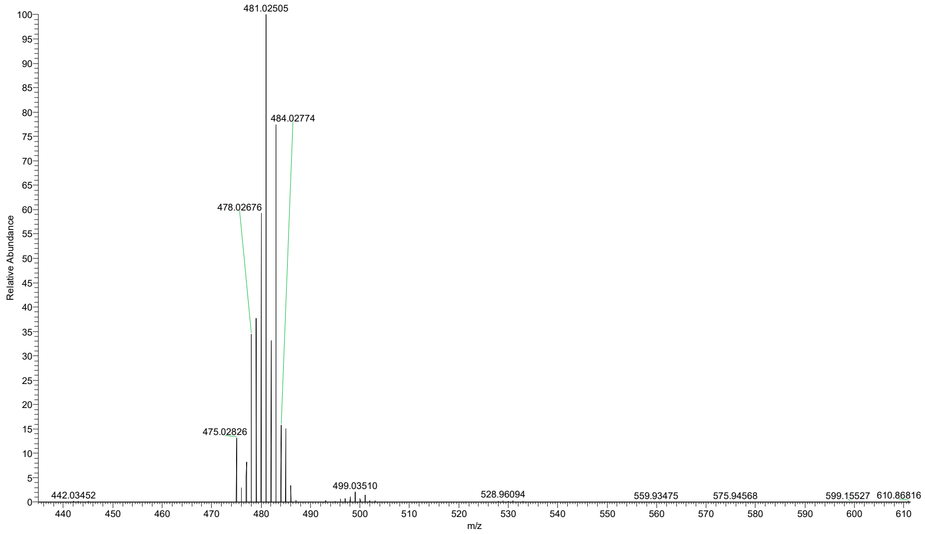

Figure S3. HRSM spectra of JHOR10.

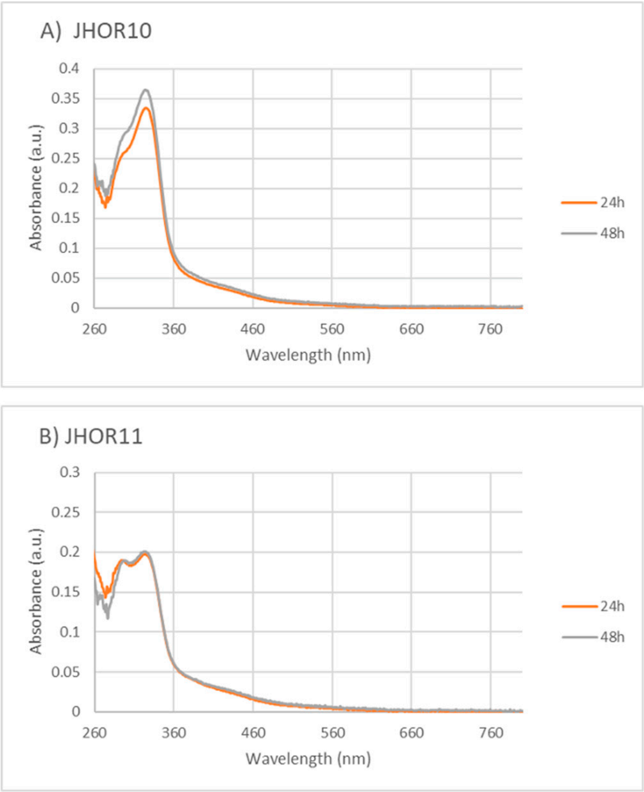

Figure S4. UV-visible spectra after 24h and 48h of 37ºC incubation of 100 µM complex A) JHOR10 and B) JHOR11 in cell culture medium DMEM.

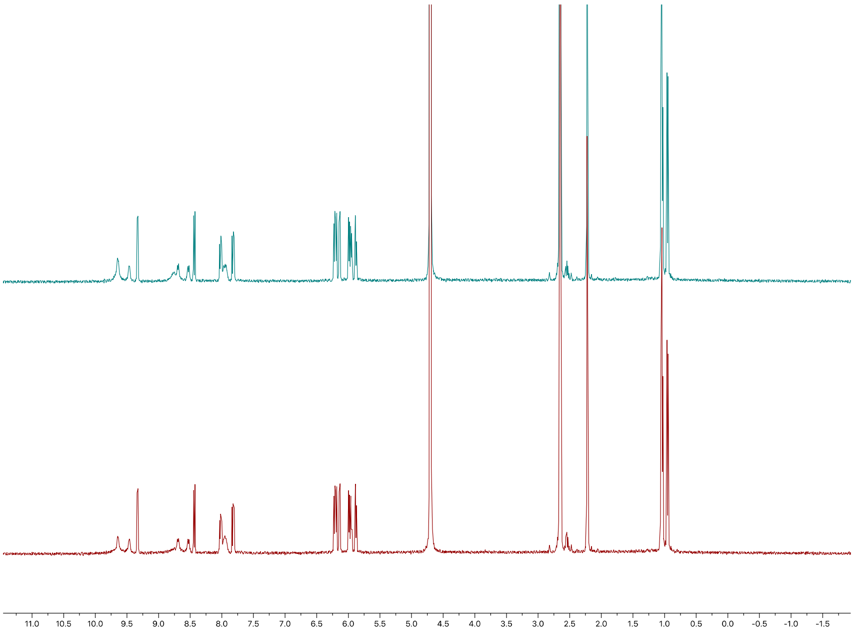

Figure S5. NMR spectra of JHOR11 in DMSO-d_6-D_2O, in a 3 : 7 (v/v) with 110 mM chloride concentration at 0h (red) and 48h (green).

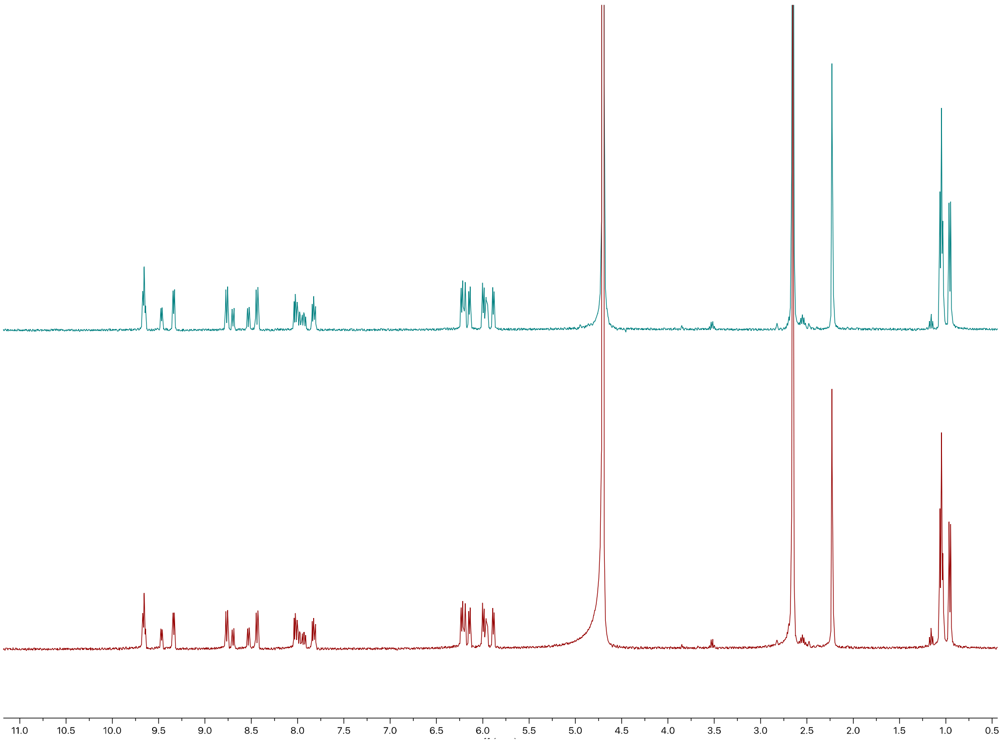

Figure S6. NMR spectra of JHOR10 in DMSO-d6-D2O, in a 3 : 7 (v/v) with 110 mM chloride concentration at 0h (red) and 48h (green).

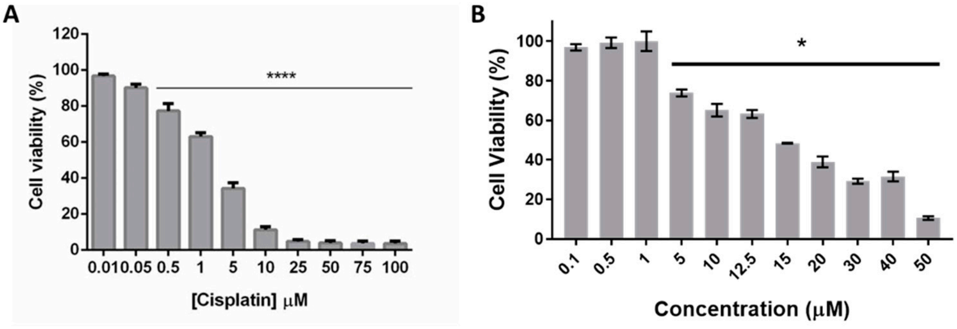

Figure S7: Antiproliferative activity of cisplatin after 48h in A) A2780 and B) HCT116 cell lines. Data was normalized to the control (0.1 % (v/v) DMSO) with bars representing the mean ± SD of at least three independent experiments. * p-value<0.05, **** p-value<0.0001.

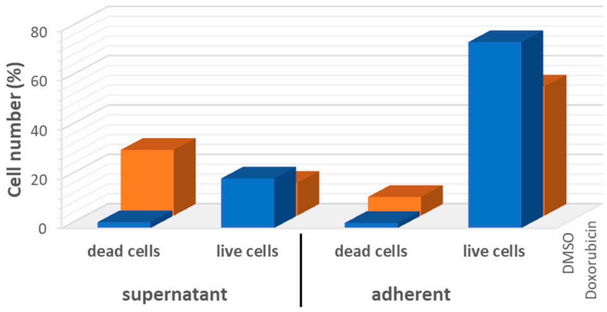

Figure S8: Percentage of live and dead A2780 cells in supernatant or adherent cells after 48h incubation with 0.1 % (v/v) DMSO, vector control, or 0.1 μM doxorubicin, for positive control.
